# Analysis and discussion on the pharmaceutical centralized procurement implementation — a case study of a large provincial hospital in China

**DOI:** 10.3389/fphar.2024.1379595

**Published:** 2024-06-03

**Authors:** Jingjing Cao, Yuan Ren, Chenglong Zhao, Li Wang, Fengqin Fang

**Affiliations:** Department of Pharmacy, Henan Provincial People’s Hospital, Zhengzhou University People’s Hospital, Zhengzhou, Henan, China

**Keywords:** centralized procurement of drugs, quantity of centralized procurement tasks, rules of centralized procurement, drug supply, repayment

## Abstract

Unaffordable medical treatment and inflated drug prices have become a challenging issue for lawmakers worldwide. To reduce the financial burden and standardize the pharmaceutical market, the Chinese government has issued several detailed regulations, including the measures of drug recruitment and procurement in one and volume purchasing to not only ensure the high quality of approved drugs but also lower the cost of the production and sell procedure. In this work, to have a thorough overview of the enforcement of these regulations, we attempted to critically analyze the data of our hospital’s centralized procurement of drugs from 2019 to 2022. We identified some concerns, such as the difficulty in determining the “quantity” of drug procurement, out-of-stock of collectively procured drugs, difficulty in managing the preallocation of associated funds, incomplete centralized procurement systems, etc. Therefore, it is essential to promote a multidimensional strategy, including the combination of the medical insurance reform and drug centralized procurement policies, strict controlling of the forecast quantity of drugs to ensure stable drug supply, improvement of the relevant policies for retaining the surplus of centralized procurement drug medical insurance funds, secureness of the drug procurement system platform, and available reference and guidance for subsequent centralized quantity procurement of drugs.

## 1 Introduction

State-organized centralized procuring of medicines with quantity, also referred to as “national centralized procurement” or “procuring with quantity”, involves the state selecting generic medicines varieties that have passed the consistency evaluation for quality and efficacy, and concentrating the scattered purchasing volume of medical institutions nationwide into a “package”. In this mechanism, the state will represent all medical institutions in negotiating prices with pharmaceutical companies.

Under the premise of strictly guaranteeing quality, this strategy may help 1) realize quantity-based procurement of drugs, 2) exchange quantity for price, 3) significantly reduce the inflated prices of medicines, and 4) effectively alleviate the financial burden on patients. The Pilot Program for State-Organized Centralized Procuring of Medicines was considered and adopted at the meeting of the Central Committee for Comprehensively Deepening Reform in November 2018 (GOV, 2023a). This program explicitly declares that it adopts the model of state organization, alliance procurement, and platform operation. It also implements bidding and centralized procurement of medicines with a determined procurement volume. Subsequently, the National Healthcare Security Bureau and the National Healthcare Commission have issued measures to deploy the national centralized procurement pilot program, implementation requirements, and regulatory programs (GOV, 2023b; GOV, 2023c; GOV, 2023d; Joint Procurement Office of China, 2018). As a populous province, Henan Province actively responded to the call of the state. On 5 December 2019, the Henan Provincial Health Insurance Bureau and eight other departments jointly issued the notice on the Implementation of the Implementation Plan for the Pilot Expansion of the Centralized Procurement and Use of Medicines by State Organizations in Henan Province (Henan Provincial Medical Protection Bureau, 2021), The notice clearly outlined that the province should comprehensively launch the implementation of the pilot expansion and the various supporting policies before December 31.

Our hospital officially implemented the results of the first batch of nationally organized drug centralized procurement selection on 31 December 2019. As of 31 December 2022, our hospital has implemented seven batches of state procurement work, one batch of provincial procurement work, and six batches of alliance centralized procurement, totalling 14 batches and 20 cycles. This study takes the 14 batches of centralized drug procurement in our hospital as the observation point and summarizes the gains and losses to provide empirical references for further improving the centralized drug procurement policy.

## 2 Materials and methods

### 2.1 Data source

Our hospital’s utilization data consists of 14 batches of collectively procured medicines during the agreement period of 2019–2022, namely, National Procurement First Batch (2 cycles), National Procurement Second Batch (3 cycles), National Procurement Third Batch (2 cycles), National Procurement Fourth Batch (2 cycles), National Procurement Fifth Batch (1 cycle), National Procurement Sixth Batch (1 cycle), National Procurement Seventh Batch (1 cycle), Henan Alliance First Batch (2 cycles), Guangdong Provincial Alliance First Batch (1 cycle), Chongqing Alliance First Batch (1 cycle), Yuzhong Alliance First Batch (1 cycle), Chinese patent drug Alliance First Batch (1 cycle), Thirteen Provincial Alliance First Batch (1cycle), and Fourteen Provincial Alliance First Batch (1 cycle).

### 2.2 Statistical method

The utilization data of medicines were retrieved through our hospital’s Hospital Information System, and the data were compared and analyzed using Excel software.

## 3 Results

### 3.1 Involvement of our hospital in drug procurement from 2019 to 2022

The cycle of centralized drug procurement is one, two, or 3 years, and the agreed purchase quantity of each drug is determined based on the actual usage in previous years. The medical insurance department requires that each medical institution fulfil the agreed purchase quantity within the cycle and have an annual evaluation of the medical institution. Medical institutions may face penalties for unfinished drugs. Table 1 shows the involvement of our hospital in drug procurement from 2019 to 2022.

**TABLE 1:** Involvement of our hospital in drug procurement from 2019 to 2022.

Time	Batch of centralized procurement of drugs	The number of selected drugs for centralized procurement	Average price reduction (%)	Maximum price reduction (%)	Completion rate of selected varieties as of 31 December 2022 (%)
31 December 2019	National Procurement First Batch	22	59.00	98.00	95.45
29 April 2024	National Procurement Second Batch	25	53.00	93.00	100.00
18 November 2020	National Procurement Third Batch	41	53.00	95.00	88.46
29 April 2021	National Procurement Fourth Batch	35	52.00	96.82	88.89
1 March 2021	Henan Alliance First Batch	21	70.71	98.91	64.28
1 October 2021	National Procurement Fifth Batch	49	56.00	98.00	76.00
1 October 2021	Chongqing Alliance First Batch	12	79.20	97.90	83.33
1 December 2021	Yuzhong Alliance First Batch	4	83.26	93.56	75.00
1 January 2022	Guangdong Provincial Alliance First Batch	45	43.00	98.00	60.00
30 May 2022	Fourteen Provincial Alliance First Batch	14	48.58	89.64	78.57
30 May 2022	National Procurement Sixth Batch	19	48.00	74.00	94.74
30 May 2022	Chinese Patent Drug Alliance First Batch	16	42.27	82.63	73.33
15 July 2022	Thirteen Provincial Alliance First Batch	36	40.00	80.29	44.44
1 October 2022	National Procurement Seventh Batch	45	48.00	98.22	31.11

The utilization rate of selected drugs in the use of drugs with the same generic name and the same dosage form in our hospitals from 2019-2022.

### 3.2 The utilization rate of selected drugs in the use of drugs with the same generic name and the same dosage form in our hospitals from 2019-2022

During the procurement cycle, medical institutions shall prioritize acquiring and utilizing the selected drugs after reaching the agreed procurement quantity. Among them, the utilization rate of the selected drugs shall not be less than 50% of the drugs with the same generic name and the same dosage form in the state procurement. The proportion of use of the selected drugs shall not fall below 80% or 70% of the drugs with the same generic name and the same dosage form in the joint procurement. Table 2 shows the utilization rate of selected drugs in the use of drugs with the same generic name and the same dosage form in our hospitals from 2019-2022.

**TABLE 2:** The utilization rate of selected drugs in the use of drugs with the same generic name and the same dosage form in our hospitals from 2019-2022.

Time	Batch of centralized procurement of drugs	Number of selected drugs for centralized procurement	Percentage of use required (%)	Number of varieties completed	Completion rate as of 31 December 2022 (%)
31 December 2019	National Procurement First Batch	22	50	22	100.00
29 April 2024	National Procurement Second Batch	25	50	25	100.00
18 November 2020	National Procurement Third Batch	41	50	41	100.00
29 April 2021	National Procurement Fourth Batch	35	50	33	94.29
1 March 2021	Henan Alliance First Batch	21	80	20	95.24
1 October 2021	National Procurement Fifth Batch	49	50	49	100.00
1 October 2021	Chongqing Alliance First Batch	12	80	12	100.00
1 December 2021	Yuzhong Alliance First Batch	4	80	3	75.00
1 January 2022	Guangdong Provincial Alliance First Batch	45	70	40	88.89
30 May 2022	Fourteen Provincial Alliance First Batch	14	80	13	92.86
30 May 2022	National Procurement Sixth Batch	19	50	19	100.00
30 May 2022	Chinese Patent Drug Alliance First Batch	16	80	14	87.50
15 July 2022	Thirteen Provincial Alliance First Batch	36	80	31	86.11
1 October 2022	National Procurement Seventh Batch	45	50	45	100.00

Our hospital’s involvement in the practice of centralized procurement of drugs.

## 4 Our hospital's involvement in the practice of centralized procurement of drugs

### 4.1 Standardized and orderly organizational management

Our hospital attaches great importance to the centralized and quantity-based procurement of drugs. We have set up a leading group for the centralized procurement and use of medicines in state organizations, with the hospital leadership heading the group to coordinate the work of centralized procurement of drugs. It has successively issued a series of documents, including the “Notice of Henan Provincial People’s Hospital on Issuing the Implementation Plan for the Centralized Procurement and Use of National Organized Drugs” (Provincial Hospital Letter [2020] No. 103), the “Notice of Henan Provincial People’s Hospital on Implementing the Measures to Ensure the Centralized Procurement and Use of National Organized Drugs” (Provincial Hospital Letter [2020] No. 104) and the Notice of Henan Provincial People’s Hospital on Issuing the 2022 Key Performance Indicator Assessment Plan (Provincial Hospital Letter [2022] No. 42), clearly led by the Department of Pharmacy, with collaboration from multiple departments such as healthcare and medical insurance. The smooth progress of centralized procurement work will be comprehensively promoted (Dong et al., 2021; Wang et al., 2021) from various aspects such as task allocation, supply guarantee, daily monitoring, dynamic control, information construction, and performance evaluation.

### 4.2 Strictly and efficiently implement policies

#### 4.2.1 Reporting the quantity of centralized procurement of drugs

According to the requirements of the relevant documents on the procurement volume of each batch of centralized procurement, our hospital calculates and reports the procurement volume data of each batch of centralized procured drugs based on the existing drug supply catalogue varieties and clinical actual drug demand. The reported quantity data is jointly discussed by relevant clinical units, medical departments, medical insurance offices, pharmaceutical departments, and other departments. The data is reviewed by hospital leaders before being reported. There is no situation where centralized procurement of drugs is not reported quantity, not purchased, or reported quantity without purchasing (He et al., 2022).

#### 4.2.2 Procurement and utilization of centralized procurement of drugs

Following relevant policy requirements, our hospital will incorporate the selected drugs from centralized procurement into the drug supply catalogue of medical institutions, strengthen the drug supply guarantee, ensure that the purchase quantity of selected drugs during the agreement period is not lower than the purchase quantity of non selected drugs with the same generic name, and prominently mark the selected drugs from centralized procurement in the prescription information system, to facilitate the clinical priority of selecting the selected varieties from centralized procurement. As of 31 December 2022, our hospital has completed seven batches of national procurement, one batch of provincial procurement, and six batches of alliance procurement, with a total of 14 batches and 19 cycles involving 384 varieties of selected drugs. Among them, only one batch of selected varieties of rabeprazole enteric-coated tablets, alanyl-glutamine injection and compound glycyrrhizic acid diamine capsule in the batch of provincial procurement has not completed the agreed procurement volume by the end of the task cycle, with the completion progress of 86.24%, 77.46% and 73.95%, respectively. The remaining selected medicines have met the agreed procurement quantity on time. As of 31 December 2022, 224 specifications of our current centralized procurement of drugs had achieved the target progress, while 81 specifications had not, resulting in an overall compliance rate of 73.44%.

#### 4.2.3 Monitoring and management of centralized procurement of drugs

Our hospital has established a dynamic monitoring mechanism for the utilization of centralized procurement drugs. Which monitors and notifies the use of selected drugs, non-selected drugs with the same generic name, and substitutable drugs of the same kind every month. Additionally, it dynamically adjusts the control measures according to the completion of centralized procurement drugs. The control measures generally include providing regular feedback to clinicians on the use of centralized procurement drugs, alerting the information system of centralized procurement drugs whose completion progress is not up to the standard, restricting the procurement and use of non-selected drugs with the same generic name and similar substitutable medicines, and convening a meeting to promote the use of non-selected drugs (He et al., 2022).

#### 4.2.4 Performance assessment of centralized procurement of drugs

The hospital incorporates the use of centralized procurement of drugs into the performance assessment of medical staff, formulates detailed performance assessment programs for centralized procurement of drugs, and implements assessment measures monthly to encourage medical staff to reasonably prioritize the use of centralized procurement of drugs (He et al., 2022).

#### 4.2.5 Repayment of drugs for centralized procurement

Following the guidelines outlined in the document “Notice on the Implementation of Health Insurance Supporting Measures for the Pilot Expansion Work of Centralized Purchasing and Use of Drugs by State Organizations” (Henan Medical Insurance Office (2019) No. 53) of Henan Provincial Healthcare Security Administration, our hospital strictly implements the provision of full payment of drugs by the end of the month following the date of delivery and acceptance. As of 31 December 2022, our hospital has made a total payment of 252 million yuan for centralized procurement of drugs (Ou et al., 2024).

#### 4.2.6 Procurement of failed bid opening and fully replaceable drugs

Following the requirements of the document “Notice on Matters Relating to the Reference Monitoring Scope of Substitutable Drugs of National and Provincial Centralized Banded Purchasing Varieties” (Henan Medical Insurance Office [2021] No. 64) issued by the Medical Protection Bureau of Henan Province, medical institutions that require the use of fully replaceable drugs within the monitoring scope and province’s centralized procurement failed bid opening drugs are required to explain the reasons in the medical record data, and failure to provide reasons will result in non-payment by the medical insurance fund. At present, our hospital supplies niacin tablets and succinimidyl gelatin injections. From 1 March 2021, to 31 July 2021, the procurement volume of niacin tablets was 0 tablets, and the procurement volume of succinimidyl gelatin injection was 15,538 bottles, with a procurement amount of 1,107,700 yuan. From 1 March 2022, to 31 December 2022, the procurement volume of niacin tablets was 0 tablets, while succinimidyl gelatin injection’s procurement volume was 7,180 bottles, amounting to RMB510,700,000. This represents a decrease of 53.79% in procurement volume and 53.90% in procurement amount compared to the corresponding period last year. As of 31 December 2022, the procurement amount for fully replaceable drugs was 53.384 million yuan. Our hospital has a monitoring mechanism for fully replaceable drugs and will implement dynamic control measures for medicines that have abnormal growth and impede the advancement of centralized procurement work.

### 4.3 Procurement of high-priced drugs

Our hospital strictly implements the price policy issued by Henan Provincial Public Resource Trading Center-Pharmaceutical Procurement, ensuring timely implementation of price linkage. As a result, there has been no procurement of high-priced drugs.

### 4.4 Procurement following the law and standardized behaviour

Drugs entering medical institutions are procured offline in strict accordance with the process of Henan Provincial Public Resource Transaction Center-Pharmaceutical Procurement Platform (Provincial Platform). For some of the drugs that are not listed on the network but must be used by patients, such as injectable botulinum antitoxin, etoposide injection and thymosin alpha-1 for injection (Ri da xian), etc., the hospital is following the Notice on the Development of the 2019 Annual Price Linkage Work of Pharmaceuticals in Henan Province (Henan Pharmaceutical Union Office) (Yu Drugs Office) [2019] No. 2) requirements, for the record offline procurement. In 2021 and the first half of 2022, 10 offline procurements of drugs in our hospital procurement amounted to 644,319.29 and 318,541.90 CNY, respectively. In 2022, the original offline procurement of drugs etoposide injection has been resumed in the provincial platform in the listing of the network, tincture of iodine [20 mL (2%)], iodine glycerine [20 mL (1%)] and Benzalkonium Chloride Patch (Bondi) [100 posts] have been discontinued in our hospital.

## 5 Discussion

### 5.1 The untimely supply of some centralized procurement of drugs

The 1-5 batches of nationally procured medicines implemented have 11 specifications with untimely supply, among which Duloxetine hydrochloride enterosoluble capsule and Entecavir tablets are continuously out of stock. The first batch of Rabeprazole tablets procured by the Henan Province were out of stock for nearly 2 months. Additionally, 45 drug varieties of Guangdong Province Union Collective Procuring were implemented from 1 January 2022. According to the requirements of the document of the Bureau of Medical Insurance, the medical institutions should release the procurement plan at the end of December 2021. Due to the maintenance of drug information on the trading platform and the delayed confirmation of electronic drug procurement and sales contracts by manufacturers and distributors, some drugs could not be submitted to the trading platform until mid-January, and a considerable portion of drugs could not be supplied on time due to the lack of stock by the distributors. As a result, only 25 medicines were delivered in the first half of January, and three medicines were not delivered until February. The sixth batch of insulin special collection was implemented at the end of May 2022; the selected medicine, “Novozymes 50 refills”, could not be delivered due to the late delivery, which triggered complaints from patients in our hospitals.

### 5.2 Multiple factors lead to difficulties in completing tasks for some centralized procurement of drugs

#### 5.2.1 Force majeure effects

Affected by COVID-19, the public awareness of personal protection has been enhanced, resulting in a reduction in respiratory diseases. As a result, the structure of patients has changed; in the first half of 2021, the amount of the commonly used pediatric respiratory drug, montelukast chewable tablets (5 mg), was only 22.24% of the amount used for the whole year in 2020.

#### 5.2.2 Medical insurance adjustment and payment method change

Since 2020, the medical insurance catalogue has been frequently updated, and the payment conditions for some varieties have changed, such as the medical insurance payment restriction for the oral normal-release dosage form of glucosamine has been changed from “paid by the basic medical insurance co-ordination fund following the regulations when used by the insured in hospitalization, and by the individual account of employees’ basic medical insurance when used in outpatient clinics” to “As our hospital is not a designated medical institution for work-related injuries in Henan Province, to a certain extent, it can be considered that the drug has been changed from a medical insurance-covered drug to a self-financed drug in our hospital, resulting in a sharp decrease in the use of the drug, for example, the use of Glucosamine Tablets in 2020 decreased by 38.31% as compared with 2019. In addition, Perindopril tert-butylamine tablets have seriously affected the completion of the centralized procurement task due to the lack of a medical insurance payment code in the early stage.

#### 5.2.3 Update of drugs and diagnostic and treatment guidelines

With the continuous updating of the concept of disease treatment, the continuous emergence of new drugs and new dosage forms, and the public’s general attention to drug safety, some of the collected varieties have been gradually replaced by better therapeutic drugs, resulting in the poor progress in the completion of such collected varieties, such as Captopril Tablets, Domperidone Tablets, and Metformin Flat Tablets.

#### 5.2.4 Difficulty in substitution of some original research drugs

Some non-selected varieties are originator drugs, which are more difficult to replace. Additionally, some of the drugs are less effective in patients’ reactions than the originator drugs. For example, antitumor drugs like Anastrozole, Imatinib, etc., have a certain degree of continuity in the clinical treatment, making it more difficult to replace them.

#### 5.2.5 Changes in centralized procurement rules

Unlike the previous centralized procurement rules, Guangdong Alliance’s centralized procurement is based on quality regulations for quantity reporting. If the reported quantity is the original research drug, which is not selected in the end, the reported quantity of the original research drug should be allocated to other drugs. Due to patients’ and clinicians’ high recognition of the original drug, allocating the quoted quantity to other drugs will put great pressure on completing the task.

### 5.3 Frequent adjustments of specifications and prices of centralized procurement of drugs

#### 5.3.1 Abnormalities in selected prices

**FIGURE 1:**
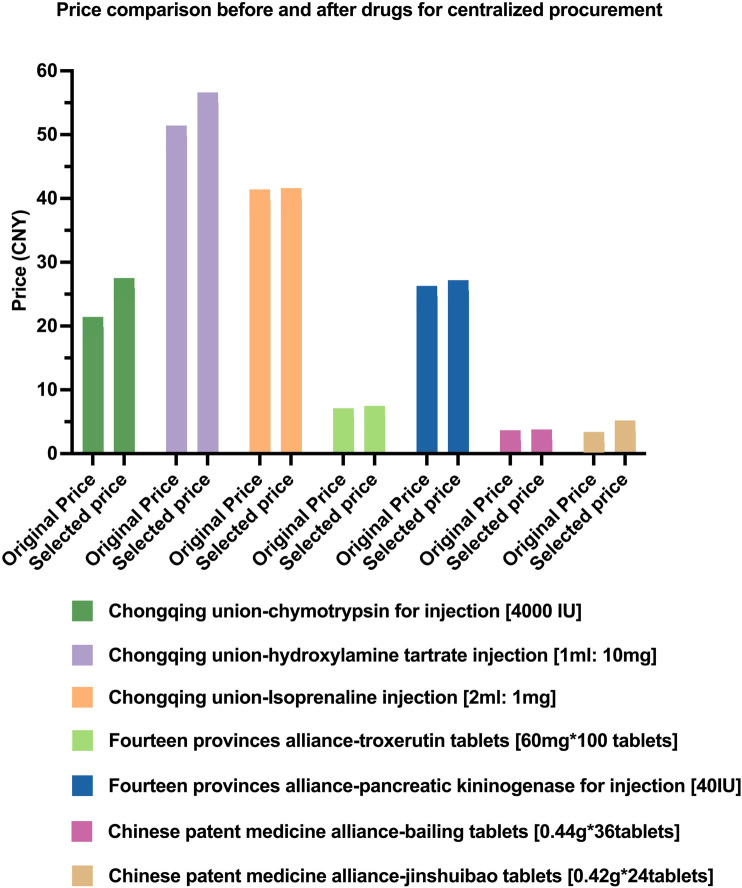
Price comparison before and after drugs for centralized procurement.

Centralized procurement of drugs is an important means to reduce the burden of medical costs on the public and save funds for medical insurance. However, in the course of implementation, it was found that the prices of some of the selected drugs increased after the selection, all of which appeared in the Union Collective Procurement. Up to now, our hospital has found that the selected price of seven drugs (such as injectable pancreatic kininogenase and injectable chymotrypsin) is higher than the original supply price of our hospital. The issue involves three batches of alliance procurement, with the specific drug varieties listed in Figure 1.

#### 5.3.2 Frequent changes in packaging

Since the implementation of centralized procurement of drugs, the specifications of the nine selected drugs signed with our hospital have changed, causing inconvenience to our hospital’s procurement and use. Among them, five drugs were unavailable due to the manufacturers’ inability to supply them, and four drugs were not included in the supply list of the selected drugs due to the specifications in use in our hospital, with the specific drug varieties listed in Table 3.

**TABLE 3:** Selected drugs for changing packaging.

Batch of centralized procurement of drugs	Drug name and current specification	Manufacturer	Selected specification
Guangdong Alliance	Clopidogrel bisulfate tablets [75 mg * 36 tablets]	Nanjing Chia-Tai Tianqing Pharmaceutical Company	75 mg*14 tablets
Guangdong Alliance	Cefuroxime axetil tablets [0.25 g*12 tablets ]	Sinopharm Zhijun (Shenzhen)Pingshan Pharmaceutical	0.25 g*24 tablets
Guangdong Alliance	Escitalopram oxalate tablets [10 mg * 21 tablets ]	Jewim Pharmaceutical (Shandong)	10 mg * 14 tablets
Fourteen Provincial Alliance	Diosmin tablets [0.45 g * 48 tablets]	Nanjing Chia-Tai Tianqing Pharmaceutical Company	0.45 g * 24 tablets
Fourteen Provincial Alliance	Pancreatic kininogenase tablets [120 IU * 48 tablets ]	Qianhong Bio-pharma	120 IU * 24 tablets
Hubei Provincial Alliance of Traditional Chinese Medicine	Shuanghuanglian oral liquid (children)[10 mL * 16 tubes]	Tailong Pharmaceutical (Henan)	10 mL * 12 tubes
Hubei Provincial Alliance of Traditional Chinese Medicine	Xiaojin capsules [0.35 g * 18 capsules ]	Jianmin Group	0.35 g * 12 capsules
Thirteen Provincial Alliance	Acarbose tablets [50 mg * 60 tablets]	Huadong Medicine	50 mg * 45 tablets
Thirteen Provincial Alliance	Metronidazole tablets [0.2 g * 100 tablets]	Kelun Industry Group	0.2 g * 21 tablets

^a^ The preparation time before the implementation of the document is too short.

### 5.4 The preparation time before the implementation of the document is too short

The reporting and quantity control of centralized drug procurement is an important part of the hospital’s implementation of centralized procurement work. According to the requirements of the hospital work, we need to solicit clinical advice widely, convene a special seminar, and, if necessary, convene the Pharmaceutical Council to determine the joint deliberations. Some of the centralized procurement documents were issued without fully considering the actual situation of the hospital, and only about 1 week (for example, the issuance time of the thirteen provincial alliance’s centralized procurement documents was 7 June 2022, with a deadline of June 15, including Saturdays and Sundays) was provided to carry out the relevant work, which was extremely rushed and caused great inconvenience to the implementation of the centralized procurement work.

## 6 Suggestions

### 6.1 Ensure the timely supply of centralized procurement of drugs

It is recommended that the health insurance department conduct a prior assessment and full-process supervision of the winning enterprises’ capacity to guarantee the medicine supply and establish a reasonable evaluation system for medicine distribution enterprises to ensure the quality of medicines and the timeliness of distribution (Wang et al., 2024). For enterprises that have experienced long-term shortages during the agreement period for various reasons seriously affecting the progress of task completion, it is recommended that they be interviewed. Such incidents are recorded in the enterprise’s record of breach of trust and urged to resume supply as soon as possible, while appropriately reducing or eliminating the number of hospital tasks (Liu, 2023; Wang and He, 2023; Zhang et al., 2023; Zhou et al., 2023).

### 6.2 Adjust the number of centralized procurement tasks appropriately under exceptional circumstances

For challenges in completing centralized procurement tasks caused by uncontrollable factors such as epidemic and disaster situations, medical insurance adjustments, and updates to drugs and diagnosis and treatment plans, it is recommended that the hospital’s workload be appropriately reduced according to the actual situation.

### 6.3 Understand the rules of centralized procurement in advance

At present, centralized procurement work has entered the stage of normalization and institutionalization. Due to the specific requirements of different batches of centralized procurement work varying, it is recommended that the health insurance department inform the hospital of the particular requirements for executing centralized procurement work when reporting the quantity of this batch of centralized procurement. This will facilitate better execution of the centralized procurement work, starting from early-stage comprehensive planning.

### 6.4 No arbitrary changes of the specifications and prices in the centralized procurement of drugs

Filling in the forecast quantity and the subsequent implementation of the centralized procurement work shall not arbitrarily change the specifications and prices of the centralized procurement drugs. For changes in the specifications and prices of centralized procurement drugs affecting the hospital to carry out centralized procurement work, the hospital should have the right to refuse to carry out the varieties of centralized procurement work and place the relevant enterprises on a blacklist.

### 6.5 Standardize the workflow to ensure that hospitals have sufficient time to carry out centralized procurement work

The Provincial Medical Insurance Bureau shall, following the standard workflow, convey the documents related to the collection and procurement to the hospital office and forward them to the relevant departments by the hospital office after the approval of the hospital leaders. It shall fully consider the actual situation of the hospital and allow sufficient time to avoid the time of issuance of the documents and the deadline of the relevant work being close to each other to enable the hospitals to make adequate preparations for a better implementation of the collection and procurement work (Liu, 2023; Zhang et al., 2023).

### 6.6 Promote the informatization of the drug procurement platform, improve the drug procurement system and its query function

At present, the province’s drug procurement system is imperfect, and relevant data information is not updated promptly, resulting in inconsistencies between the delivery quantity and the inbound quantity of drugs when medical institutions and medical insurance departments check the procurement situation of drugs; Secondly, on the drug procurement platform, only the cumulative purchase quantity, delivery quantity, and inbound quantity of drugs can be found, and it is not possible to display the monthly or each drug procurement and actual delivery situation of medical institutions. Therefore, promoting the informatization of the drug procurement platform can facilitate medical institutions and health insurance departments in accurately querying the procurement information of centralized procurement drugs. Additionally, adding the module of the value of the advance payment of collectively procured drugs purchased by each medical institution to the drug procurement platform can simplify the workflow of each department (Weng et al., 2022; Li et al., 2023).

## Data Availability

The original contributions presented in the study are included in the article/Supplementary material, further inquiries can be directed to the corresponding author.
